# First evidence of circulation of multiple arboviruses in Algeria

**DOI:** 10.1371/journal.pntd.0012651

**Published:** 2024-11-07

**Authors:** Saïd C. Boubidi, Laurence Mousson, Tahar Kernif, Fayez Khardine, Aïssam Hachid, Cécile Beck, Sylvie Lecollinet, Rayane A. Moraes, Sara Moutailler, Catherine Dauga, Anna Bella Failloux

**Affiliations:** 1 Institut Pasteur d’Alger, Eco-Epidémiologie Parasitaire et Génétique des Populations, Alger, Algeria; 2 Institut Pasteur, Université Paris Cité, Arboviruses and InsectVectors, Paris, France; 3 Laboratoire des Arbovirus et Virus Emergents, Institut Pasteur d’Algérie, Algiers, Algeria; 4 Faculté de Pharmacie, Université d’Alger1, Algiers, Algeria; 5 ANSES, INRAE, Ecole Nationale Vétérinaire d’Alfort, UMR Virologie, Laboratoire de Santé Animale, Maisons-Alfort, France; 6 ANSES, INRAE, Ecole Nationale Vétérinaire d’Alfort, UMR BIPAR, Laboratoire de Santé Animale, Maisons-Alfort, France; NIAID Integrated Research Facility, UNITED STATES OF AMERICA

## Abstract

**Background:**

Algeria like other North African countries is experiencing recurrent episodes of West Nile Virus (WNV) emergences and new health threats associated with the introduction of *Aedes albopictus* in 2010 are to be feared. To improve the surveillance of mosquito-borne pathogens, we performed a study using innovative tools based on multiplex molecular methods.

**Methods:**

We combined two approaches: a high-throughput chip based on the BioMark Dynamic array system to detect arboviruses in mosquitoes, and a set of immunologic methods (ELISA, microsphere immunoassays (MIA) and virus microneutralization tests (MNT)) for serological surveys in animal hosts. We investigated two distinct regions: a first zone located in the coastal humid region and a second one in the Saharan desert region.

**Principal findings:**

We collected a total of 1,658 mosquitoes belonging to nine different species and found predominantly *Culex pipienss*. *l*. (56.5%) and *Cx*. *perexiguus* (27.5%). From 180 pools of 10 mosquitoes, we detected four arboviruses: Banna virus (BAV), chikungunya virus (CHIKV), Sindbis virus (SINV), and Usutu virus (USUV). Moreover, we examined 389 blood samples from equids and poultry and found that 52.4% were positive for flavivirus antibodies in ELISA, while 30.8% were positive for WNV and two chickens and two equids were positive for USUV by MNT and MIA respectively.

**Conclusions:**

To our knowledge, this is the first report of five arboviruses circulating in Algeria, with three reported for the first time (CHIKV, BAV, and USUV). Our study brings evidence that reinforcing surveillance using more discriminant tools may help in anticipating future emergences and propose adapted control measures.

## Author summary

Arboviruses are a significant threat to public health because of their epidemic and zoonotic potential. The geographical distribution of mosquito-borne diseases such as West Nile Fever has expanded over the last decades. Countries of the Mediterranean region are not spared. In this context, surveillance of the circulation of such viruses needs to be achieved before the appearing of human or animal cases. The aim of our study was to use innovative and highly efficient tools to highlight the circulation of arboviruses in mosquito vectors, animal reservoirs or exposed animals in Algeria. A total of 1,658 mosquitoes belonging to nine different species were collected and screened for arboviruses usinghighthroughput chips based on the BioMark Dynamic arrays system, capable of screening various arboviruses in a single experiment. Moreover, we analyzed 389 blood samples from equids and poultry using 3 different serologic methods. Our results bring, for the first time in Algeria, evidence of the cryptic circulation of 5 arboviruses: West Nile virus, chikungunya virus, Usutu virus, Sindbis virus and for the first time out of the Asian region, Banna virus.

This approach, as an early warning system, is enabling for an early detection of pathogens circulation above-threshold before disease appearance in humans.

## Introduction

Algeria as the largest country in Africa (2 381 741 km^2^) covers different bioclimatic zones from humid to Sahelian presenting a high level of biodiversity. It experienced outbreaks of different arthropod-borne diseases such as malaria [1,2], leishmaniasis [3], plague [4] and rickettsiosis [5]. With 53 species described [6], mosquitoes constitute an important group of vectors, transmitting malaria [2] and multiple arboviruses [7–10]. Among arboviruses, West Nile virus (WNV) was first isolated from a pool of *Culex* spp. mosquitoes in 1968 in Djanet in the south-east of Algeria [7]. Later, in 1994, a WNV epidemic was reported in Tinerkouk oasis in the south-west of Algeria, with 50 human cases and eight deaths mainly among children [11]. Neighboring countries also experienced WNV outbreaks, Tunisia in 1997 (111 human cases, 8 fatalities) [12] and Morocco in 1996 and 2010 (horse encephalitis) [12,13]. Later, in 2012, a retrospective serosurvey in Algiers and surrounding areas highlighted specific anti-WNV IgG in 11 out of 164 human samples tested [14]. During the same year, a fatal neuro-invasive case was reported for the first time in northern Algeria, in the province of Jijel [15]. In 2018, the WNV lineage 1 was detected in a pool of *Cx*. *perexiguus* collected in Timimoun [8].

While equids and humans are dead-end hosts with short-lasting and low level viraemia that do not allow mosquitoes infection [16], birds (resident and migrating) are resistant and play the role of reservoirs [17,18]. Migratory birds pass through Algeria coming from sub-Saharan Africa, Middle East and Europe and stopover in wetlands. Passerines are considered the main competent hosts for WNV and Usutu virus (USUV), a closely genetically related virus [19]. Some wild birds, and specifically passerids, raptors and Charadriiforme aquatic birds generally develop a viraemia greater than 10^5^ PFU/mL, capable of infecting *Culex* vectors [18,20,21]. WNV and USUV, two flaviviruses belonging to the *Flaviviridae* family, share eco-epidemiologic features and transmission cycles. During WNV outbreaks in New York City in 2000, tens of thousands of birds of 54 native bird species died, mostly crows [22, 23]. USUV has been shown to infect 62 bird species derived from 18 orders and 26 families [24]. Sentinel birds, like chickens and pigeons have been used for decades to monitor arthropod-borne virus circulation [25]; they develop antibodies after infection without becoming highly infectious to *Cx*. *pipiens* vectors [24]. Globally, WNV and USUV surveillance includes the detection of clinical cases in humans and equids (horses and donkeys), as well as programmed serological surveillance in equids in addition to survey in resident and migratory birds, and the viral screening of mosquitoes [26].

Several *Culex* mosquito species are involved in WNV and USUV transmission in wild or captive avifauna [25] and in the transfer to susceptible mammals in particular, humans (WNV, USUV) and horses (WNV) [27]. *Cx*. *pipiens*, a species feeding on birds and humans, is considered to be the main vector in Europe [20]. In Algeria, WNV was detected in *Culex spp*. [7] and particularly, *Cx*. *perexiguus* [8]. In 2017, four pools of *Cx*. *perexiguus* and six pools of *Cx*. *pipiens* were found infected with Sindbis virus (SINV) [10]; this alphavirus of the *Togaviridae* family, causes human diseases in Africa, Europe, Asia, and Australia [28]. Like WNV and USUV, the primary vectors of SINV are ornithophilic *Culex (Cx*.*)* mosquitoes [28].

*Culex*-transmitted arboviruses including migratory birds as hosts have been circulating in the region, including Algeria, for a long time [9]. Regarding the increasing number of WNV foci in the world, the situation may have evolved in Algeria, especially knowing that most of infected cases are asymptomatic or present Flu-like symptoms and then are misdiagnosed. In this study, we investigated the current situation of arboviruses circulation among vectors and reservoirs in Algeria. What has changed is the frequency of emergences and the number of human cases, most likely related to intensive urbanization creating the conditions for mosquitoes to proliferate in close proximity to birds and humans [29]. Using highly sensitive tools, we investigated the circulation of arboviruses in collected mosquitoes in two different bioclimatic zones of Algeria. A novel high throughput surveillance method has been designed using a microfluidic system (BioMark dynamic array system, Standard Biotools) capable of performing parallel real-time PCRs using 96.96 chips resulting in 9,216 individual reactions and detecting 95 different genotypes/serotypes of 37 viral species [30]. Additionally, serological analysis based on IgG detection on blood samples of equines and poultry was performed using three complementary methods: cELISA, MNT and MIA.

## Methods

### Ethics statement

The Institute Pasteur of Algeria has received accreditation from the Algerian Ministry of Health and Agriculture to perform experiments and blood sampling on live animals in compliance with the World Animal Health Organization guiding principles on animal welfare included in the OIE Terrestrial Animal Health Code [31]. This study was approved by the Institutional Animal Care and Use Committee (IACUC) at the Institute Pasteur of Algeria. Verbal informed consents were obtained from animal owners and director of El Kala Zoo prior to the collection of blood samples.

### Study area

**Fig 1:**
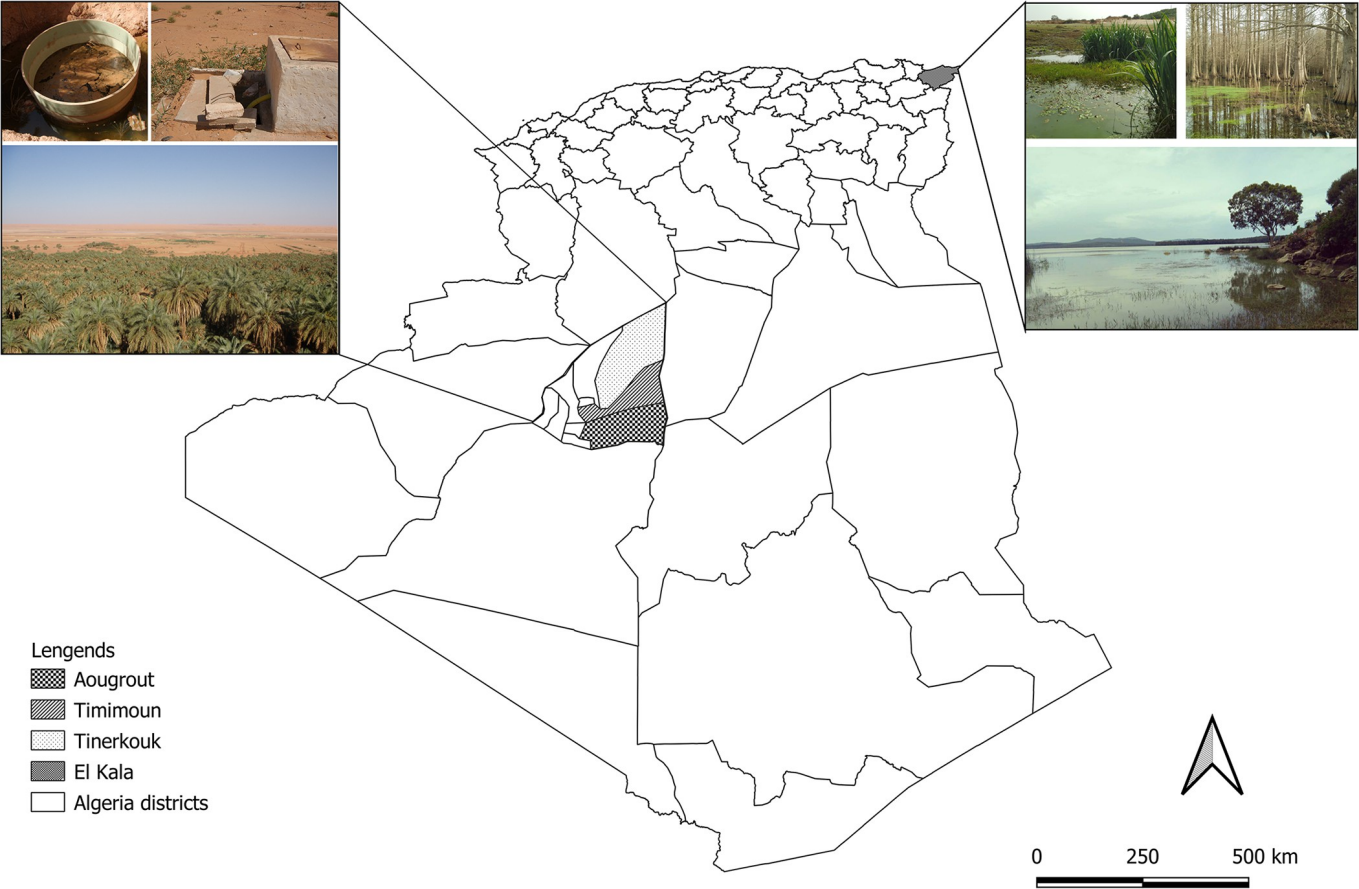
Map of mosquito and animal (equids and birds) collection sites. The map was built using the open source map site: Qgis 3.28.13 Software(https://qgis.org/license/), Shape files: Algeria subnational administrative boundaries: https://data.humdata.org/dataset/cod-ab-dza?https://data.humdata.org/dataset/3d17274d-0812-4b21-b87a-4854af4eb244/resource/34f317e5-7de9-4e1b-a457-91ab786dc952/download/dza_admbnda_unhcr2020_shp.zip.

Study sites were selected based on documented circulation of WNV in the country [11,32]. Thereby, two sites with episodic proven WNV circulation were chosen: (i) El Kala in the coastal humid region at El-Tarf department (36°53’N, 8°26’E; rainfall of 900–1200 mm/year) and (ii) Tinerkouk (30°17’N, 0°56’E), Timimoun (29°12’N, 0°24’E) and Aougrout (28°50’N, 0°59’E) in the Saharan desert region at Adrar department (rainfall of 100 mm/year) (Fig 1).

In each bioclimatic region, different habitats were chosen: urban (center of the city), sub-urban (outskirts of the city) and rural (outside the city). This mosquito sampling strategy was implemented to be exhaustive in collecting all species of mosquitoes present in the studied sites.

### Mosquitoes

Adult mosquitoes were collected inside animal shelters and human habitations during four consecutive nights using eight CDC light traps (John W. Hock, Gainesville, FL) baited with carbon dioxide dry ice in July and October 2018 and October 2019 (from 7.00 pm to 7.00 am). These collecting periods corresponded to the period of maximum mosquito activity (highest densities) in Algeria. Caught mosquitoes were identified alive on ice using the morphological keys of Brunhes et al. 2000 [33]. Females with the presence of blood meal in their abdomens were not taken into account for viral screening. Once identified, specimens were immediately dissected by separating the abdomen from the remaining part of the body (RPB). RPB screening was carried out for two reasons: i) to identify the infected mosquitoes in each positive pools to estimate the infectious rate and ii) to identify the probably competent vector for the detected arbovirus.

Ten abdomens from the same species were pooled in 1.5 mL Eppendorf tubes. The corresponding RPB were placed individually in 0.2 mL Eppendorf tubes. Both parts were stored rapidly in liquid nitrogen container until arriving to the laboratory where they were stored at -80°C until RNA extraction.

### RNA extraction

Total RNAs were extracted from each pool using the Nucleospin RNA II extraction kit (Macherey-Nagel, Hoerdt, France). Pools were ground in 600 μL of L15 medium with 20% fetal calf serum (FCS) using an homogenizer (Retsch, Germany) operating at 30 Hz/s for 3 min. After centrifugation at 7,000 rpm for 6 min at +4°C, total RNA per pool was eluted in 50 μL of RNase free water and stored at −80°C until use. When a virus was detected in pools of abdomens, the RPB (head/thorax) of individual mosquitoes composing each pool were homogenized in 300 μL of L 15 medium with 2% FCS using the homogenizer 24 Dual (Bertin, France) at 5,500 rpm for 20 s. Then, total RNAs were extracted from 150 μL of homogenates using the Nucleospin RNA II extract kit (Macherey-Nagel, Germany) and 150 μL were stored at -80 C° for attempts to isolate the virus. Total RNA per sample was eluted in 40 μL of RNase free water and stored at -80 C° until use.

### Reverse transcription and cDNA pre-amplification

RNAs were transcribed to cDNA by reverse transcription using the qScript cDNA Supermix kit according to the manufacturer’s instructions (Quanta Biosciences, Beverly, USA). For cDNA pre-amplification, the Perfecta Preamp Supermix (Quanta Biosciences, Beverly, USA) was used according to the manufacturer’s instructions. These two procedures were performed according to the protocols described by Moutailler et al. (2019) [30].

### Arboviruses screening by High-Throughput Real-Time Microfluidic PCRs

cDNAs were screened for detection of 37 arboviruses in the BioMark Dynamic arrays system (Standard Biotools, CA, USA). Amplifications were performed using 6-carboxyfluorescein (FAM)-and black hole quencher (BHQ1)-labeled TaqMan probes with TaqMan Gene expression master mix (Applied Biosystems, France). Thermal cycling conditions were: 2 min at 50°C, 10 min at 95°C, followed by 40 cycles of 2-step amplification of 15 sec at 95°C, and 1 min at 60°C. Data was acquired on the BioMark Real-Time PCR System and analyzed using the Fluidigm Real-time PCR analysis software to obtain crossing point values. One negative water control was included per chip. To determine if inhibitors present in the sample could inhibit the real-time PCR, a strain of *Escherichia coli* was added to each sample as an internal inhibition control. Among the 149 primers/probe sets detailed in Moutailler et al. (2019) [30], 95 sets targeting 95 different genotypes/serotypes of 37 viral species were chosen for our screening (list primers/probs in S1 Additional information). Positive samples were confirmed by the iTaq Universal Probes One-Step Kit (Biorad, CA, USA). The 20 μL reaction mix contained 10 μL of iTaq universal probes reaction mix (2×), 10 μM of each primer, 3 μL of cDNA. A Biorad T100 thermal cycler (Biorad, CA, USA) was used, and the cycling conditions were: 95°C for 3 min, and 40 cycles at 95°C for 10 s, 60°C for 20 s. Once the detection of virus is confirmed by qRT PCR, using specific primers and probes listed in S1 Table, a screening of corresponding individual RBP was performed by real-time PCR.

### Viral serodiagnosis

#### Animal blood sampling

In total, 389 sera were collected from equids and poultry during October 2018 and October 2019. Equids, belonged to domestic horses and donkeys, and sampled poultry to domestic chickens and ducks, and exotic birds in El Kala zoo, such as peacocks, pheasants and guinea fowls from the El Kala zoo. During our study, we did not sample any exotic animals in the Saharan sites because there was no zoo in Timimoun nor in Tinerkouk. Blood samples were collected from the jugular vein of horses and the ulnar vein of poultry, centrifuged for separation of serum and stored at +4°C or -20°C until being processed. All sampled equines were healthy and were not vaccinated against WNV.

All sera were first screened by cELISA to detect anti-flavivirus antibodies. Positive samples were then confirmed by virus microneutralization test (MNT) to detect WNV and USUV neutralizing antibodies specifically and the microsphere immunoassay (MIA) which allows to distinguish between WNV and USUV exposure through the binding of animal sera on beads coupled to WNV and USUV specific antigens.

#### Competitive ELISA

The serological diagnosis was initiated using a cELISA test (ID screen WNV competition ELISA kit, ID Vet, France) on poultry and equids sera samples. Interpretation was performed according to the manufacturer’s instructions. The threshold value for considering a serum as positive was % OD sample/negative control (%S/N) ≤ 40% as recommended by the manufacturer. Those with a 40% <%S/N ≤ 50% or less and greater than 40% were considered doubtful, and those with a %S/N > 50% were considered negative. This test has been used to give an indication of the presence or absence of anti-WNV antibodies in sera, but cross-reactions with other flaviviruses may occur; consequently, reactive sera were considered flavivirus-positive [34].

#### Microsphere immunoassay

Equids sera positive samples on cELISA were screened by flavivirus microsphere immunoassay (MIA) as described by Beck et al. (2015) [35]. Purified recombinant envelope domain III (rEDIII) proteins and non structural 1 (NS1) of WNV and USUV were used for the capture of specific IgG antibodies. A recombinant soluble ectodomain of WNV envelope (E) glycoprotein (WNV.sE) which identifies exposure by flaviviruses in the same way as the competitive ELISA test and WNV.EDIII, USUV.EDIII. EDIII, WNV.NS1 and USUV.NS1 were coupled to microspheres using the Bio-Plex Amine coupling kit (Biorad, CA, USA) according to the manufacturer’s instructions as described in Beck et al. (2015) [35]. Five micrograms of WNV.sE, 10μg of WNV.NS1 and USUV.NS1or TBEV.EDIII or 50 μg of WNV.EDIII and USUV.EDIII were coupled with the beads. The sera (diluted 1/100) were then tested with the MIA technology using an equimolar mixture of the five beads as previously described in Beck et al. (2015) [35]. For each antigen, the diagnostic cut off was determined from the mean median of fluorescence (MFI) values of 66 horse-negative sera plus 3 standard deviations of the mean. In cases of positive reactions with several rEDIIIs or NS1s, an animal was considered infected with WNV if WNV.rEDIII or WNV.NS1generated an MFI at least two-fold greater than that generated with USUV.rEDIII or USUV.NS1 respectively. If a two-fold difference could not be achieved, the sample was considered to be infected with WNV or USUV. The sample was considered positive for an undetermined *Flavivirus* if it reacted with WNV.sE but not with any of the rEDIIIs or NS1s.

#### Virus microneutralization tests

All ELISA positive samples from equids and poultry were further investigated through virus-specific microneutralisation tests (MNTs) against flaviviruses reported in the area where the sera were collected. These samples were investigated through MNT against WNV and USUV for the detection of specific neutralizing antibodies against WNV (strain Is98, Genbank ID AF481864.1, provided by P. Desprès, IPP) and USUV (strain France 2018, Genbank ID MT863562.1) following the protocol described in Beck et al. (2015) [35]. A serum was considered positive if cells were protected at the 1:10 serum dilution for WNV and USUV. Owing to cross-neutralization for members belonging to the Japanese encephalitis serocomplex, such as WNV and USUV, we identified the infecting *Flavivirus*, WNV or USUV, by considering the virus with the highest neutralization capacity, and with neutralization titers that differed by at least a four-fold dilution factor [35]. If this latter condition was not met, the serum was considered positive for WNV or USUV.

### Sequencing and phylogenetic analysis

Primers were designed with primer design program Primer 3 to target the segment 12 of the BAV genome: BaV-S12-P5-for (5’-TGTGGGTTGTGAGGGTCCAA-3’) and BaV-S12-P5-rev (5’-AGTAGCATA AGATGCATGGCG-3’). Classic PCR reactions were performed in a final volume of 25 μL containing 1.5 mM of MgCl2, 0.2 mM of each deoxyribonucleoside triphosphate (dNTPs) mixture, 1× PCR buffer, 0.05 U/μL of Taq DNA Polymerase, 0.2 μMof each primer, and 5 μL of genomic DNA. The PCR reactions were run in a Biorad T100 thermal cycler (Biorad, CA, USA)using the following reaction conditions: 95°C for 3 min for initial denaturation, 40cycles at 95°C for 30 s, 58°C for 30 s, and 72°C for 1 min, and a final extension at 72°C for 5 min. The PCR products were subjected to electrophoresis in a 1.5% agarose gel stained with ethidium bromide and then visualized using UV light.

The 12^th^ segment genes of 40 BAV strains and four other representatives of Seadornaviruses (Banna-like virus, Kadipiro virus, Liao ning virus and Mangshi virus) were selected based on samples used in previous studies [36,37].

Multiple sequence alignment, phylogenetic analysis based on the maximum likelihood method, and the general time reversible (GTR) model were conducted, using MEGA v10.2.6 [38]. All positions with less than 90% of site coverage were eliminated, i.e. fewer than 10% alignment gaps, missing data, and ambiguous bases were allowed at any position (partial deletion option). There was a total of 666 positions in the final dataset. Initial tree(s) for the heuristic search were obtained automatically by applying Neighbor-Joining and BioNJ algorithms to a matrix of pairwise distances estimated using the maximum composite likelihood (MCL) approach, and then selecting the topology with superior log likelihood value. A discrete Gamma distribution was used to model evolutionary rate differences among sites (5 categories (+G, parameter = 4.8623)).Statistical support of phylogenetic tree obtained was assessed by bootstrap analysis with 100 replicates and bootstrap values for each node are given if > 50%.

In addition, the patristic or phyletic distances within and between taxa of BAV groups were calculated. Pairwise patristic distances correspond to the amount of genetic change between any two sequences as depicted by the branch lengths in a phylogenetic tree.

## Results

### Screening of arboviruses in mosquitoes

A total of 1,658 mosquitoes belonging to six genera and nine species collected in the four study sites were processed. 910, 452, 275 and 21 female mosquitoes were collected in El Kala, Timimoun, Tinerkouk and Aougrout respectively (S1 Fig; S2 Table). Nine species were identified; the most abundant species were *Cx*. *pipiens* sl. (56.5%) and *Cx*. *perexiguus* (27.5%), the remaining females were *Aedes caspius* (9.3%), *Anopheles labranchiae* (2.2%), *Coquillettidia richiardii* (1.8%), *Uranotaenia ungiculata* (1.3%), *Cq*. *buxtoni* (1.1%), *Culiseta longiareolata* (0.2%) and *An*. *d’thali* (0.1%) (S1 Fig).

The 1,658 field-collected mosquitoes were grouped into 180 pools for viral screening. Viral detections using the BioMark microfluidic system showed the presence of four arboviruses: CHIKV, USUV, SINV and Banna Virus (BAV) (S2 Fig and Table 1). Three pools from El Kala province, urban *Cx*. *pipiens*, rural *Cx*. *perexiguus* and rural *An*. *labranchiae*, were found positive for CHIKV. Moreover, two pools of *Cx*. *pipiens* from urban habitat of El Kala province were found positive for BAV, whilst one pool of *Cx*. *perexiguus* from rural habitat of El Kala province and one pool of *Cx*. *pipiens* from urban habitat from Timimoun province were found positive for USUV. One pool of *Cx*. *perexiguus* originating from Tinerkouk province was found positive for SINV. When analyzing RPB (head-thorax) of individual mosquitoes, we found one USUV-infected mosquito, one SINV-infected mosquito and five BAV-infected mosquitoes. No RPB were infected in the three positive CHIKV pools.

**Table 1:** Viruses detected using high-throughput chip based on the BioMark Dynamic arrays system in mosquitoes collected in different districts of Algeria (2018–2019).

Collection sites					Mosquitoes				Viruses			
District	Province	GPS coordinates	Climate	Habitat (number of CDC traps)	Species	Number of mosquitoes	Number of pools (% of positive)		Virus detected using microfluidic system (Ct value)	Confirmation by RT-PCR	Virus detection in RBP by RT-PCR	Confirmation by sequencing
El Taref	El Kala	36°53’N, 8°26’E	Humid	Urban (2)	*Cx*. *pipiens*	280	28 (10.7%)		CHIKV (Ct 25)	(-)	(-)	/
									BAV (Ct 18)	(+) Ct 17	(+) Ct 17.5 (+/-) Ct 30 (+/-) Ct 35	(+)
												/
												/
									BAV (Ct 25.6)	(+) Ct 29	(+/-) Ct 36 (+/-) Ct 36	/
												/
				Rural (3)	*Cx*. *perexiguus*	418	50 (4%)		CHIKV (Ct 25)	(-)	(-)	/
									USUV (Ct 15.6)	(+) Ct 23	(+) Ct 27.2	/
					*An*. *labranchiae*	26	3 (33.3%)		CHIKV (Ct 25)	(+) Ct 35	(-)	/
Adrar	Timimoun	29°12’N, 0°24’E	Arid	Urban (2)	*Cx*. *pipiens*	315	32 (3.1%)		USUV (Ct 19)	/	(-)	/
	Tinerkouk	30°17’N, 0°56’E		Peri-urban (3)	*Cx*. *perexiguus*	8	1 (100%)		SINV (Ct 22.7)	(+) Ct 34	(+) Ct 25.4	/
	Aougrout	28°50’N, 0°59’E		Peri-urban	0	0		0	0	/	/	/

RBP, remaining body part (head and thorax); Ct, cycle threshold.

/ = not performed

Two pools among 28 of *Cx*. *pipiens* from urban site of El Kala province tested positive for BAV, representing 5 positive mosquitoes for an infection rate of 1.8% (5/280), whilst one of 50 pools of *Cx*. *perexiguus* from rural site of El Kala province and one of 32 pools of *Cx*. *pipiens* from urban site from Timimoun province were found positive for USUV. However, the unique pool of *Cx*. *perexiguus* originating from Tinerkouk province was found positive for SINV. When analyzing RPB (head-thorax) of individual mosquitoes, we found one USUV-infected mosquito, one SINV-infected mosquito and five BAV-infected mosquitoes representing an infection rate of 0.2% (1/418), 12.5% (1/8) and 1.8% (5/280) respectively. No RPB were infected in the three positive CHIKV pools, although detected, it was probably due to positive blood meal as Culex is not a suspected vector.

For BAV, one pool (29K18) consisted of three positive RPBs with lowest cycle threshold (Ct) values of 17.5, 30 and 35 and the second one (30K18) revealed two positive RPBs with higher Ct values: 36 and 36. The RNA extract from *Cx*. *pipiens* RPB showing a high detection level (Ct: 17.5) was reverse-transcribed to cDNA and amplified by classic PCR. The obtained amplicon of 600 bp length was sequenced by Eurofins (Nantes, France), using Sanger sequencing system, and blasted confirming the BAV (GenBank acc no. OQ305924).

### Serology

In total, 389 sera were collected and first screened by cELISA to determine WNV seropositivity. Out of 204 positive samples, 111 and 93 from El Kala and the three Saharan sites respectively were found positive for flavivirus antibodies (Table 2). In El Kala, 74 of equids, including 21 horses and 53 donkeys, and 37 of birds, including 18 chickens, seven ducks, five peacocks, four pheasants and three guineas fowl were flavivirus positive. In the three Saharan sites, 25 horses were flavivirus positive in Timimoun, two horses in Tinerkouk, and two donkeys in Aougrout. Only chickens were sampled in Timimoun with 64 animals flavivirus positive.

**Table 2:** Detection of antibodies (WNV, USUV, Flavivirus) in animal sera by cELISA and MNT.

Study site	Animal species	cELISA	MNT		
		Flavivirus positive / total (%)	WNV positive / total (%)	USUV positive / total (%)	WNV or USUV positive / total (%)
El Kala	Horse (*Equus ferus caballus*)	21/25 (84%)	16/25 (64%)	0	4/25 (16%)
	Donkey (*Equus asinus*)	53/103 (51.4%)	36/103 (34.9%)	0	7/103 (6.8%)
	Chicken (*Gallus gallus domesticus)*	18/47 (38.3%)	14/47 (29.8%)	0	0
	Duck (*Anas platyrhynchos)*	7/19 (36.8%)	7/19 (36.8%)	0	0
	Peacock *(Pavo cristatus)*	5/9 (55.5%)	1/9 (11.1%)	0	4/9 (44.4%)
	Pheasant (*Phasianus colchicus*)	4/7 (57.1%)	2/7 (28.6%)	0	2/7 (28.6%)
	Guineafowl *(Numida meleagris)*	3/4 (75%)	2/4 (50%)	0	0
Timimoun	Horse (*Equus ferus caballus)*	25/37 (67.6%)	18/37 (48.6%)	0	0
	Donkey (*Equus asinus)*	/	/	/	/
	Chicken (*Gallus gallus domesticus*)	64/129 (49.6%)	21/129 (16.3%)	2/129 (1.5%)	33/129 (25.6%)
Tinerkouk	Horse (*Equus ferus caballus)*	2/7 (28.6%)	2/7 (28.6%)	0	0
Aougrout	Donkey (*Equus asinus)*	2/2 (100%)	1/2 (50%)	0	0

All ELISA-positive samples were screened by MNT (Table 2).WNV-specific antibodies were identified by MNT in the majority of flavivirus-exposed animals. In El Kala, 52 equids, including 16 horses and 36 donkeys, and 26 birds, including 14 chickens (seven ducks, one peacock, two pheasants, two guineas fowl were found WNV positive. In the three Saharan sites, 18 horses and 21 chickens in Timimoun, two horses in Tinerkouk, and one donkey in Aougrout were WNV-positive. Interestingly, we found two chickens from Timimoun USUV-positive by MNT (Table 2).

MIA using EDIII marker showed that in El Kala site, 15 of horses and 25 of donkeys were WNV positive antibodies, and one horse and one donkey were USUV positive antibodies. Moreover, we found that four horses and 12 donkeys were WNV or USUV positive antibodies, and 11 donkeys were undetermined flaviviruses positive antibodies (Table 3). In Timimoun, we showed that 17 of horses were WNV positive antibodies, three WNV or USUV positive antibodies and three undetermined flaviviruses positive antibodies. In Tinerkouk, only two horses were WNV positive antibodies (Table 3).

**Table 3:** Specific detection of WNV and USUV antibodies against EDIII and NS1 in animal sera by MIA.

Study site	Species	MIA (EDIII)				MIA (NS1)			
		WNV	USUV	WNV or USUV	Flavivirus	WNV	USUV	WNV or USUV	Flavivirus
El Kala	Horse (*Equus ferus caballus*)	15 (60%)	1 (4%)	4 (16%)	0	15 (60%)	1(4%)	3 (12%)	0
	Donkey (*Equus asinus*)	25 (24.2%)	1 (1%)	12 (11.6%)	11 (10.7%)	45 (43.6%)	0	3 (2.9%)	3 (2.9%)
Timimoun	Horse (*Equus ferus caballus)*	17 (45.9%)	0	3 (8.1%)	3 (8.1%)	17 (45.9%)	0	0	5 (13.5%)
Tinerkouk	Horse (*Equus ferus caballus)*	2 (28.6%)	0	0	0	2 (28.6%)	0	0	0
Aougrout	Donkey (*Equus asinus*)	0	0	0	0	0	0	0	0

MIA using NS1 protein confirmed MIA results obtained with EDIII-coated beads, generally identifying WNV exposure in cELISA-positive horses (El Kala:15, Timimoun:17, Tinerkouk: 2). Moreover, in donkeys, WNV exposure were more efficiently confirmed by MIA with NS1 than with EDIII: 45 donkeys in El Kala were found infected by WNV, three by WNV or USUV and three by another flavivirus with NS1-MIA, while25 animals only were WNV-positive, 12 were found infected by WNV or USUV and 11 by another flavivirus in EDIII.MIA (Table 3). The percentage calculated was based on total sample numbers, assuming all cELISA negative would also be negative by MNT and MIAs.

### Taxonomic status of Banna virus from Algeria

To clarify the taxonomic status of Banna virus from Algeria, similarity values and phylogenetic relationships between sequences of the 12^th^ segment of the isolate BAV-like-Algeria-2018 (OQ305924) and other BAVs were analyzed.

Similarity values of OQ305924 with nucleotide sequences of BAVs from different genotypes were low, from 80.34% to 81.37% with type A1, from 80.17% to 82.08% with type A2, and from 81.71% to 81.88% with type B (S3 Table). Similarity values were even smaller with MH521275 and QX884648 sequences of the type C, 77.4% and 79.07%, respectively.

**Fig 2:**
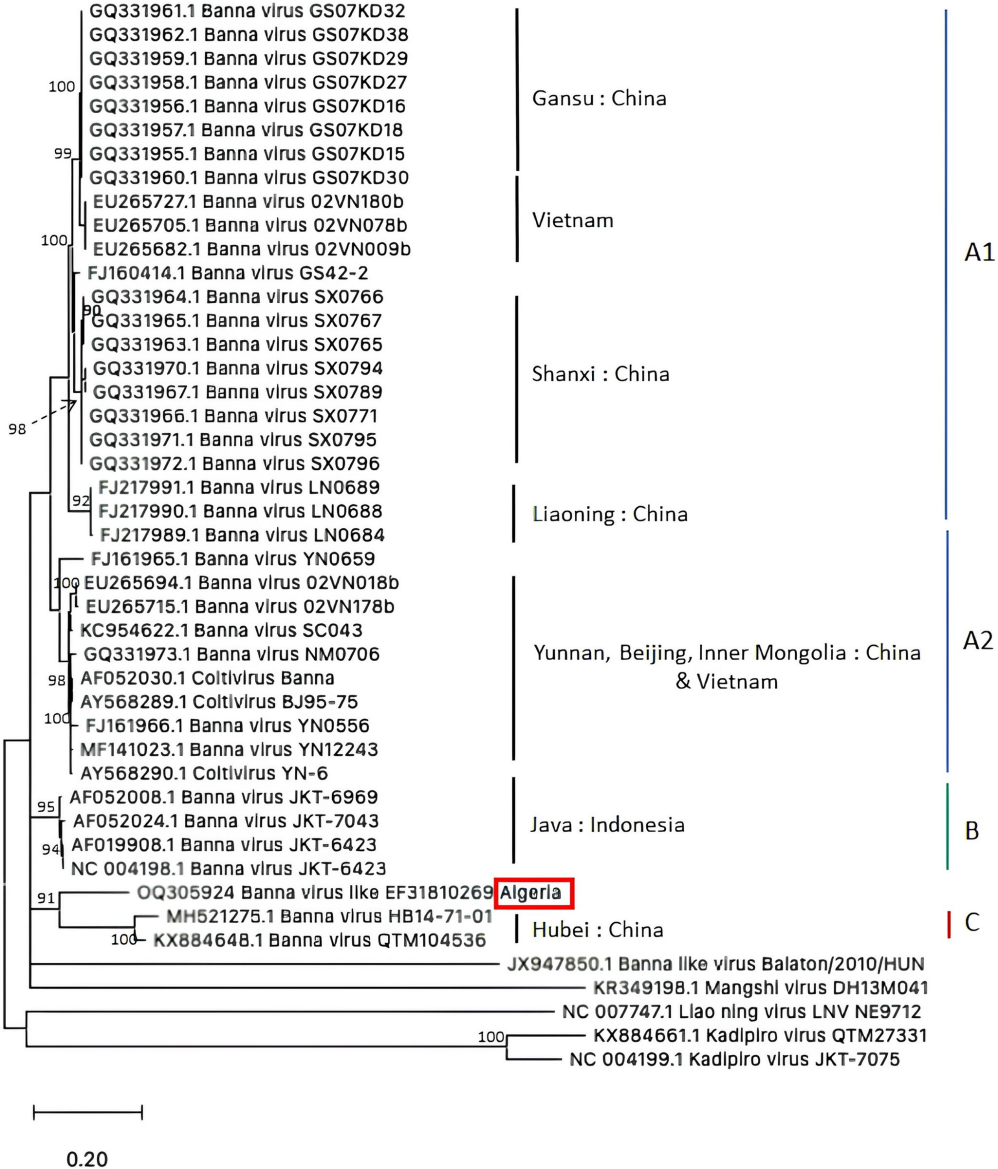
Phylogenetic tree of Banna virus based on the 12^th^ segment (SV12). Sequences were aligned by MUSCLE. The phylogenetic tree was inferred using the Maximum Likelihood method, the General Time Reversible model and bootstrap method. The tree with the highest log likelihood (-6246.98) is shown.

The maximum likelihood (ML) tree revealed that our sequence clustered together with segment 12 of Banna viruses (Fig 2). The most closely related group of BAVs with our sequence was the group (genotype) C, represented by viruses isolated from *An*. *sinensis* in Hubei, China. Their relationship was supported by 92% bootstrap as shown in the Fig 2 (Fig 2). However, patristic distances between our sequence and the 12^th^ segment of BAVs of group C (GTR dist = 0.42) remain much higher than pairwise distances within and between the other groups (S4 Table). These results suggest that the virus, BAV-like-Algeria-2018 isolated from *Cx*. *pipiens* in Algeria, formed a separate branch from other previously reported BAVs.

## Discussion

To our knowledge, this is the first report of co-circulation of multiple arboviruses in Algeria. Indeed, our study highlighted the circulation of five different arboviruses in the studied sites, BAV, CHIKV, SINV, USUV, and WNV. Our investigations were focused on sites where the circulation of arboviruses had been documented many years ago, and specifically WNV, as evidenced by high anti-WNV seropositivity detected in equids (42%) and in domestic poultry (21.9%). After analyzing animal sera with competitive flavivirus ELISA, we improved our screening using more specific serological methods, namely MNT and MIA, which allowed to confirm that flaviviruses such as WNV and USUV are circulating in Algeria, probably for many years. Despite the non-reporting of WNV clinical cases in humans or equids, our investigations on 389 animal samples showed that 52.4% (204/389) were cELISA positive indicating an important circulation of Flaviviruses at El Kala and Saharan studied sites; 111 (54.4%) and 93 (45.6%) from El Kala and the three Saharan sites respectively (see Table 2); MNT analysis indicated that WNV was the main flavivirus circulating in both sites.

Globally, flavivirus seropositivity for equids and poultry were 59.2% (103/174) and 47% (101/215) respectively which are significantly higher than in other neighboring countries such as in Morocco (33.7% in horses) [39] and in Iran (16.66% in equids and 13.75% in birds) [40]. In 2014, Lafri et al. (2017) [32] showed that in El Kala, out of 222 donkeys and 41 horses, 16 (39%) horses and 32 (14.4%) donkeys were WNV-antibodies positive, showing that WNV circulation has intensified since then. Likewise, from November 2014 to February 2015, Medrouh et al. (2020) [19] showed that in Kabylia, north-center of Algeria, 12.5% and 1.5% of song thrushes (*Turdus philomelos*) and house sparrows (*Passer domesticus*) had been exposed to WNV, these values are much lower than seropositivity in birds detected in our study (between 36.8% and 75%; Table 2).

Despite high seroprevalences, there were no clinical cases detected among tested birds. Repetitive contacts with WNV introduced from migratory birds could have led to an acquired immunity of domestic and exotic encaged birds. This scenario is different comparing the one in North America in 1999 where WNV originated from Israel [41] caused an important mortality among native birds, especially Corvids, Sparrows and even exotic animals in New York City [42]. Millions of birds died from WNV infection and population declines of >50% have been observed for several bird species [43].

Interestingly, anti-USUV antibodies were detected by MNT in two chickens in the Timimoun site. This is the first report of USUV in Algeria. Our results show the presence of two USUV-positive (1.5%) chickens from Timimoun and MIA helped at evidencing two USUV-positive (1.5%) equids, one horse and one donkey from El Kala site. Medrouh et al. (2020) [19] found that in Kabylia region, north central of Algeria, WNV was circulating among wild birds (7.8%) but not USUV. This virus first identified in south Africa in 1959 by Mcintosh (1985) [44], was reported in several other African countries in addition to Algeria: Central African Republic, Senegal, Ivory Coast, Nigeria, Uganda, Burkina Faso, Tunisia and Morocco [45].

We used three complementary techniques to detect flaviviruses, cELISA, MNT, and MIA. cELISA is the first-line method because of its ease of execution. MNT is the gold standard assay for the confirmation of exposure by flaviviruses as it identifies antibodies neutralizing a specific flavivirus. However, MIA proved to be more informative than MNT when combining EDIII and NS1 antigens. Indeed, the flavivirus MIA, which was developed for the detection of exposure to flaviviruses in horse sera proved to be more sensitive than MNT, recognizing 6 more exposure to WNV and 2 USUV exposure in equids (one positive horse and one positive donkey for EDIII-coupled beads and the same positive horse using NS1-coupled beads). This method needs more developments to be used on avian species and other arboviruses [34]. The flavivirus MIA protocol needs improvements for serological diagnosis of flaviviruses: using a wider range of proteins and including nonstructural proteins (NS) such as NS1 or NS5 to distinguish vaccinated from naturally infected animals or recent from old infections. MIA can be developed for flaviviruses circulating in other animal species [35].

We combined serological surveys with entomological investigations. We used a newly developed high-throughput virus-detection assay based on microfluidic real-time PCRs able to detect 64 mosquito-borne viruses in mosquitoes [30]. Out of 180 pools analyzed, eight pools of mosquitoes were found positive for four different species of arboviruses. We detected BAV in two pools of *Cx*. *pipiens* in urban El Kala, CHIKV in three pools of mosquitoes (*Cx*. *pipiens* in urban El Kala, *Cx*. *perexiguus* in rural El Kala, and *An*. *labranchiae* in rural El Kala), SINV in one pool of *Cx*. *perexiguus* in peri-urban Tinerkouk and USUV in two pools (*Cx*. *perexiguus* in rural El Kala and *Cx*. *pipiens* in urban Timimoun). The four viruses belong to three different families, USUV to *Flaviviridae* (like WNV), CHIKV and SINV to *Togaviridae* and BAV to *Reoviridae*.

The detection of a virus in mosquitoes does not infer on their capacity to transmit this virus through mosquito bite. Vector competence for WNV is well documented for *Culex* species: *Cx*. *antennatus*, *Cx*. *pipiens*, and *Cx*. *univittatus* in Egypt and Israel [46,47]; *Cx*. *quinquefasciatus* in India [48]; *Cx*. *univittatus*, *Cx*. *theileri*, *Cx*. *quinquefasciatus*, and *Cx*. *neavei* in South Africa [49,50]; and *Cx*. *tritaeniorhynchus* in Pakistan [51]. In Algeria, WNV was first detected in a pool of *Culex spp*. mosquitoes in 1968 in Djanet, extreme south-east of Algeria [7]. The competence of *Cx*. *pipiens* from three biogeographical sites (North, Center and South) of Algeria was demonstrated for two viruses, WNV and RVFV (Rift Valley Fever Virus) [52]. WNV lineage 1 was detected in a pool of *Cx*. *perexiguus* from Aougrout, south of Algeria [8]. Nevertheless, despite a high seropositivity of WNV detected in equids and chickens, we did not detect WNV in mosquitoes while CHIKV, USUV and SINV were detected in *Cx*. *perexiguus*. This vector is considered as the main vector of WNV in Egypt, Israel [53], Portugal, Italy and Spain [54,55]. *Cx*. *perexiguus* is also incriminated as a potential vector of USUV and SINV [54,56,57]. Usually, the ornithophilic species, *Cx*. *pipiens*, is considered to be the main vector of USUV in Europe [58] besides other mosquitoes (*Cx*. *modestus*, *Cx*. *neavei*, *Cx*. *perexiguus*, *Cx*. *perfuscus*, *Cx*. *quinquefasciatus*, *Cx*. *univittatus*, *Ae*. *albopictus*, *Ae*. *japonicus*, *Ae*. *minutus*, *An*. *maculipennis*, *Cs*. *annulata*, *Mansonia africana*, *Ma*. *aurites*, *Ochlerotatus caspius* and *Oc*. *detritus*) [20]. *Cx*. *pipiens*, *Cx*. *neavei* and *Cx*. *quinquefasciatus* are experimentally competent to transmit USUV [58–60].

We detected SINV in *Cx*. *perexiguus* in Tinerkouk. Like CHIKV, SINV causes arthralgia and myalgia. It is reported in Eurasia, Africa and Australia with clinical manifestations mainly observed in northern Europe and South Africa [28]. This virus has been mainly isolated from *Cx*. *pipiens* but also *Cx*. *torrentium*, *Cx*. *univittatus*, and *Cx*. *theileri* [61]. In Algeria, in 2017, Ayhan et al. (2022) [10] reported for the first time the presence of SINV in *Cx*. *pipiens* and *Cx*. *perexiguus*; a SINV named SINV-Algeria_2017-*Cx*. *perexiguus* has been fully sequenced (GenBank accession number: OK644705). CHIKV has also been detected in *Cx*. *perexiguus* abdomens but not in head-thorax suggesting that this vector took a blood meal on a CHIKV-infected host in the El Kala site with no evidence of viral dissemination and transmission by the mosquito. CHIKV was also detected in abdomens of *Cx*. *pipiens* and *An*. *labranchiae* but not in head-thorax. CHIKV circulates actively in the northern part of Algeria and the implication of both *Culex* and *Anopheles* mosquitoes in transmission can be ruled out. On the other hand, the omnipresence of *Ae*. *albopictus* since 2010 in northern Algeria, can be the cause of future CHIKV emergences [62]. Further studies using BG sentinel traps targeting *Ae*. *albopictus* will be more informative.

Against all expectations, we detected BAV from *Cx*. *pipiens*. *s*.*l*. in urban El Kala. Originating from Asia [63], BAV has never been detected outside its natural range of distribution. RNA was detected in pools of abdomens and then, confirmed in head-thorax leading to identify five BAV-infected mosquitoes. We used strongly positive RNA extract (pool 29K18, Ct = 17.5) for sequencing.

Phylogenetic analysis shows that our strain of BAV-like detected in Algeria is part of a distinct and new branch of BAV species. Our strain is closely related to the Indonesian and Chinese BAV sequences rather than to other viruses (e.g. Balaton virus, Kadipiro virus, Liao ning virus, Mangshi virus) (Fig 2). Further studies are needed to isolate more strains of this virus in the same site and to perform whole genome sequencing to confirm that our strain belongs to a new group of BAV.

This virus has been probably introduced in El Kala by infected persons who travelled recently in Asia. Asian workers in construction and public works travel regularly between Algeria and China.

Our study emphasizes a cryptic circulation of multiple arboviruses in Algeria, first by detecting them in mosquitoes and second by serological confirmation in animals. The use of discriminant tools, the high-throughput screening arboviruses system applied for mosquitoes [64,65] and the MIA serological method for animals [34] will allow to define more precisely the circulation of arboviruses and then, to predict future emergences. Both methods can be adapted for screening more pathogens (bacteria, parasites in addition to virus) in different specimens (other taxa, additional tissues,…) [66]. Efforts should be invested in developing serological markers adapted to MIA for viral screening in birds and equines. Furthermore, our study should be extended to migratory birds for the detection and isolation of WNV and USUV. Sustained surveillance of animals and mosquitoes using discriminant tools will help to detect emergence at the earliest stages and to react quickly to contain the spread.

## Supporting information

S1 Additional information List of the 37 viruses targeted (95 primers/probe sets for different genotypes/serotypes). (DOCX)

S1 Fig Mosquito species collected in El Kala (A) and the three Saharan sites of Tinerkouk, Timimoun, and Aougrout (B).
(TIF)

S2 Fig Screening of mosquitoes collected in Algeria using the BioMark dynamic array system (96.96 chip). Each square corresponds to a single real-time PCR reaction. Each row refers to mosquito pools and each column to arbovirus targeted. Ct values for each reaction are indicated in color: the darkest shade of blue and black squares as negative reactions (Ct>37) and the lightest shade of blue and orange as positive reactions (Ct<37).
(TIF)

S1 Table Primer and probes sequences used for the confirmation of the BioMark dynamic array system positive samples. (DOCX)

S2 Table Details of identified mosquitoes collected from four provinces of Algeria. (XLSX)

S3 Table Comparison of sequence OK644705 with 12^th^ segments of BAVs from different genotypes using Blastn: Homology values and Scores. (XLSX)

S4 Table Phylogenetic distance according to the GTR+Gamma model for 12^th^ segments of BAVs and the isolate BAV-like-Algeria-2018 (OQ305924). (DOC)
