# Supplementary material for: First evidence of circulation of multiple arboviruses in Algeria
Source: PLoS Negl Trop Dis. 2024 Nov 7;18(11):e0012651. doi: 10.1371/journal.pntd.0012651 (PMC11575824; doi:10.1371/journal.pntd.0012651)
Supplement: S1 Additional information — (DOCX) [file pntd.0012651.s001.docx]

**S1 Additional information**

**Targeted viruses**

The 37 viruses targeted (95 primers/probe sets for different genotypes/serotypes) were: Banna; Banzi; Barmah Forest; Batai; Bwamba; Chikungunya (Chik_ECSA, Chik_IndECSA, Chik_Asia, Chik_Wafri, ChikV); Dengue (D1, D2 D3, D4, Deng_1, Deng_2, Den_3, Deng_4); Murray Encephalitis (EnM_gI, Enm_gII, EnM_gIII, EnM_gIV, EnM_gV); Yellow fever (YFV, FJ_AO_1, FJ_AO_3_6, FJ_AO_4, FJ_AC_AE, FJ_AmS_1, FJ_AmS_2); Germiston; Ilesha; Inkoo; Japanese Encephalitis (JEV, JEV_I, JEV_II, JEV_IIIa, JEV_IIIb, JEV_IV, JEV_V); Kedougou (KedV); Kokobera group (Kok_ I, Kok_II, Kok_Bai, Kok_Tor, Kok_Map, Kok_Strat); Koutango (Kout_I, Kout_II); Middelburg; Ndumu; Ngari; Nyando; O’nyong-nyong (ONN); Orungo; Pongola; RossRiver (RossRiver, Ross1, Ross2); Rift Valley Fever (RVFV, RVF_SegS); Saboya; Semliki forest; Simbu; Sindbis (Sindbis, Sind_I, Sind_2_3, Sind_IV, Sind_V, Sind_VI); Spondweni (Spond_I, Spond_II); Tahyna; Uganda; Usutu; Wesselsbron; West Nile (WN, WN_1A, WN_1B, WN_1C, WN_2.1, N_2.2, WN_3, WN_4); Yaounde; Zika (Zika2, Zika3, Zika4, Zika6, Zika7, Zika8).
