# Supplementary figures and images for: First evidence of circulation of multiple arboviruses in Algeria
Source: PLoS Negl Trop Dis. 2024 Nov 7;18(11):e0012651. doi: 10.1371/journal.pntd.0012651 (PMC11575824; doi:10.1371/journal.pntd.0012651)

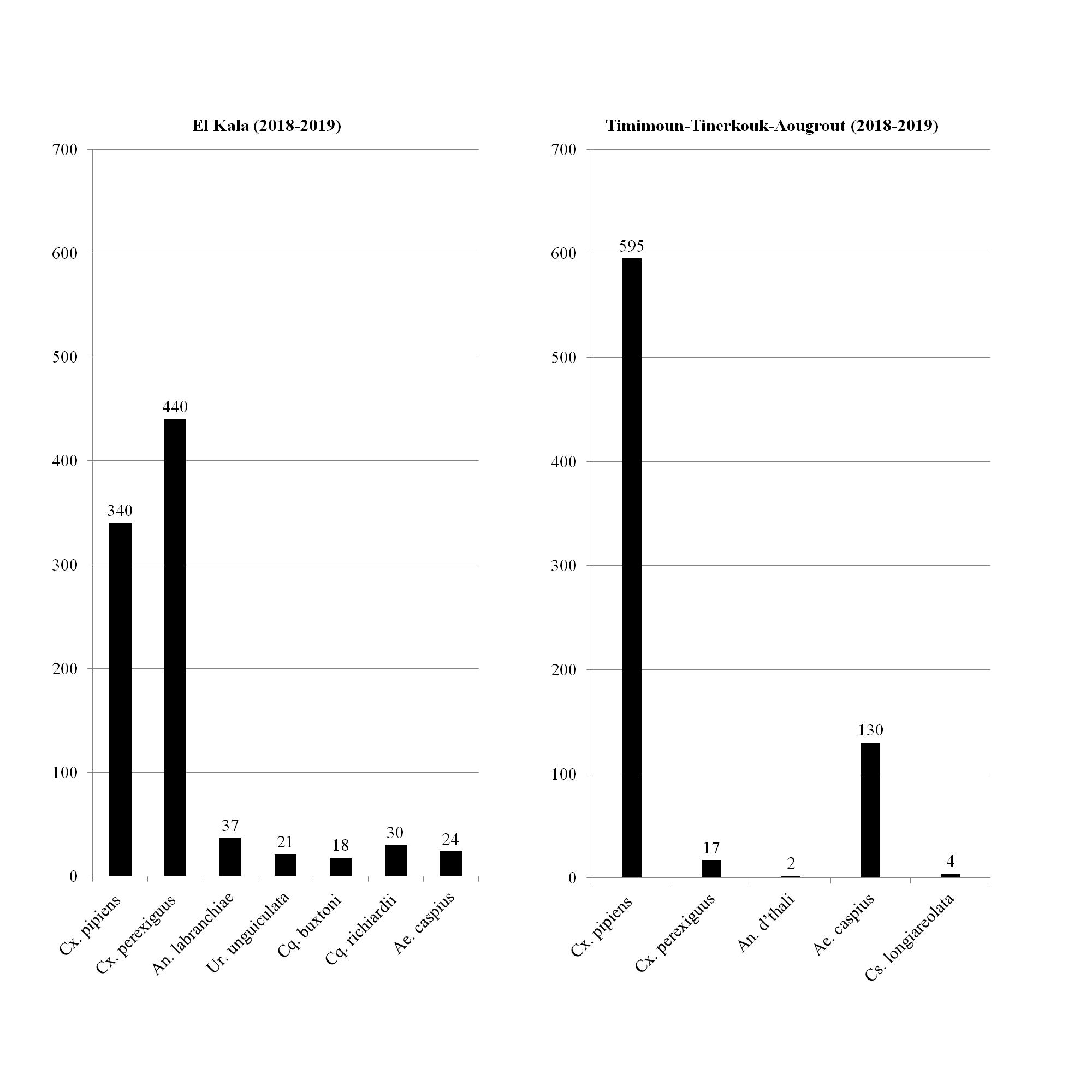

Supplement: S1 Fig — Mosquito species collected in El Kala (A) and the three Saharan sites of Tinerkouk, Timimoun, and Aougrout (B). (TIF) [file pntd.0012651.s002.tif]

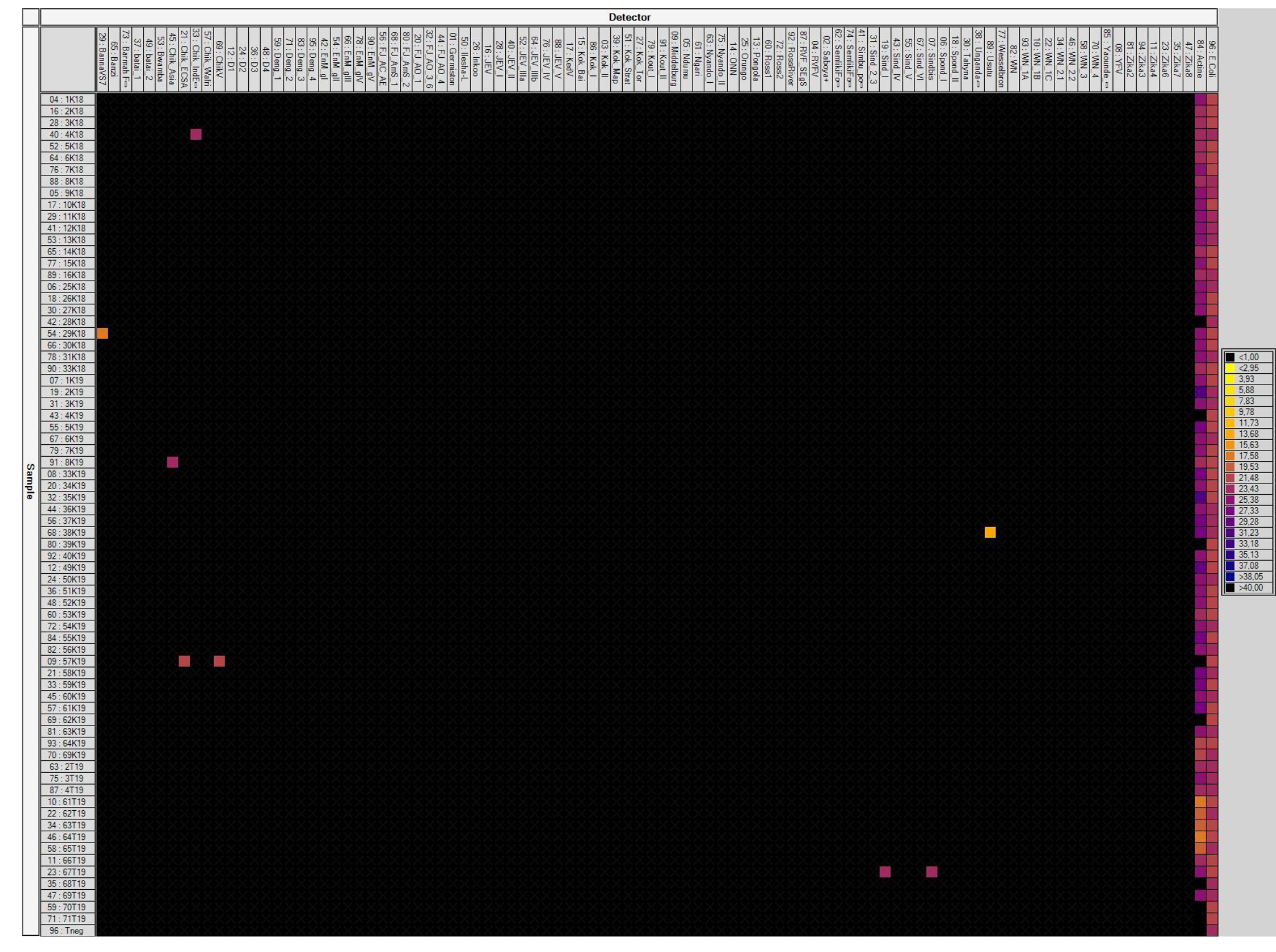

Supplement: S2 Fig — Each square corresponds to a single real-time PCR reaction. Each row refers to mosquito pools and each column to arbovirus targeted. Ct values for each reaction are indicated in color: the darkest shade of blue and black squares as negative reactions (Ct>37) and the lightest shade of blue and orange as positive reactions (Ct<37). (TIF) [file pntd.0012651.s003.tif]
