# Supplementary material for: First evidence of circulation of multiple arboviruses in Algeria
Source: PLoS Negl Trop Dis. 2024 Nov 7;18(11):e0012651. doi: 10.1371/journal.pntd.0012651 (PMC11575824; doi:10.1371/journal.pntd.0012651)
Supplement: S1 Table — (DOCX) [file pntd.0012651.s004.docx]

**S2 Table: Primer and probes sequences used for the confirmation of the BioMarkTM dynamic array system positive samples.**

| Virus | Method | Primer/prob sequences |
| --- | --- | --- |
| Chikungunyia Virus | RtqPCR (sybr green) | Chik/E2/9018 : CACCGCCGCAACTACCG |
|  |  | Chik/E2/9235 : GATTGGTGACCGCGGCA |
|  | RtqPCR (Taqman) | chik_F_SG : TGG AAT GGC TGG TTA ACA AGA TAA |
|  |  | chik_R_SG: CTC CGC GGA CAC CTA ACG |
|  |  | chik-P2: ACG GCC ACC ACG TGC TCC TGG T |
| Usutu Virus | RtqPCR (Taqman) | USUTU 550-F :  CACGCAACATGGGAAAAACC |
|  |  | USUTU 648-R :  GCATCCAGTTTGGGGCATTC |
|  |  | USUTU 574 –probe : TGCTGGATTAGAGCCATGGATGTCGGGTA |
| Banna Virus | PCR on cDNA | Bav-S12-P5For : TGTGGGTTGTGAGGGTCCAA |
|  |  | Bav-S12-P5Rev : AGTAGCATAAGATGCATGGCG |
| Sindbis Virus | qPCR on cDNA (BioradiScript) | CACCCCGCACAAAAATGAC |
|  |  | AAAAGGGCAAACAGCCAACTC |
|  |  | SinV-P :(6-Fam)-TCAAGCCGCCATCTCAAAAACATCAT-(BHQ1) |
