# Supplementary material for: First evidence of circulation of multiple arboviruses in Algeria
Source: PLoS Negl Trop Dis. 2024 Nov 7;18(11):e0012651. doi: 10.1371/journal.pntd.0012651 (PMC11575824; doi:10.1371/journal.pntd.0012651)
Supplement: S4 Table — (DOC) [file pntd.0012651.s007.doc]

**S3 Table**

Phylogenetic distance according to the GTR+Gamma model for 12^th^ segments of BAVs and the isolate BAV-like-Algeria-2018 (OQ305924).

| **BAV** | **Within groups** | **Between groups** | | | **With Algerian virus** |
| --- | --- | --- | --- | --- | --- |
|  |  | **A2** | **B** | **C** |  |
| **A1** | 0.03698461 | 0.09940352 | 0.14719085 | 0.38804023 | 0.335567913 |
| **A2** | 0.02826655 | - | 0.13382138 | 0.37538567 | 0.3219231 |
| **B** | 0.010240833 | - | - | 0.34666275 | 0.293507 |
| **C** | 0.059266 | - | - | - | 0.420592 |
